# Sleeping on a problem: the impact of sleep disturbance on intensive care patients - a clinical review

**DOI:** 10.1186/s13613-015-0043-2

**Published:** 2015-02-26

**Authors:** Lori J Delaney, Frank Van Haren, Violeta Lopez

**Affiliations:** Clinical Nursing, University of Canberra, Canberra, Australia; Australian National University, Canberra, Australia; Department of Intensive Care Medicine, Canberra Hospital, Canberra, Australia; School of Medicine and Health Sciences, Australian National University, Canberra, Australia; Yong Loo Lin School of Medicine, National University of Singapore, Singapore, Singapore

**Keywords:** Clinical care, Environment, Intensive care, Light, Sleep, Sleep deprivation, Sleep fragmentation, Noise

## Abstract

Sleep disturbance is commonly encountered amongst intensive care patients and has significant psychophysiological effects, which protract recovery and increases mortality. Bio-physiological monitoring of intensive care patients reveal alterations in sleep architecture, with reduced sleep quality and continuity. The etiological causes of sleep disturbance are considered to be multifactorial, although environmental stressors namely, noise, light and clinical care interactions have been frequently cited in both subjective and objective studies. As a result, interventions are targeted towards modifiable factors to ameliorate their impact. This paper reviews normal sleep physiology and the impact that sleep disturbance has on patient psychophysiological recovery, and the contribution that the clinical environment has on intensive care patients’ sleep.

## Review

### Introduction

Sleep is considered to be an essential biological function to maintain physiological and emotional wellbeing. However, sleep in the intensive care unit (ICU) has been reported by survivors of critical illness to be of a poor quality and a major stressor associated with their admission [1]. The proclivity for sleep and circadian disturbance amongst ICU patients has been attributed to the intrusive clinical care and clinical environment, with environmental factors inclusive of noise, and light as etiological causes. As a result, patients exhibit symptoms consistent with dyssomnia, such as difficulty with sleep initiation and fragmentation, and early morning awakenings [2]. This paper discusses the impact of environmental factors: noise, light and clinical care interactions on patient sleep and the psychophysiological consequences of sleep disturbance.

#### Normal sleep architecture

Sleep involves a number of sequential stages, with two predominate phases: non-rapid eye movement (NREM) and rapid eye movement (REM) sleep. NREM consists of three stages which constitutes between 75% to 80% of the total sleep time (TST) [3] with the first stage (N1) acting as a transition between wakefulness and deep sleep. N1 is characterised by physical drowsiness, combined with decreased ocular movements and a reduction in muscle activity [3,4]. This is followed by N2, in which the individual becomes decreasingly unaware of their surroundings, yet remains easily roused by noise [5]. As sleep progresses, N2 becomes the predominant stage making up 45% to 55% of the NREM phase.

Stage N3 consists mostly of slow wave activity and is considered to be an anabolic and physically restorative stage [6]. This stage is prominent in the first third of the night’s sleep and is deemed to be the deepest and most restful stage of the sleep cycle. During slow wave sleep (SWS), metabolic activity is at its lowest leading to a reduction in oxygen consumption. Growth hormone is secreted during this stage which promotes protein synthesis, tissue healing and physical recovery [7]. In contrast, the proceeding REM phase features increased cerebral and physiological activity, whereby the brain’s metabolic rate reflects that of a wakeful state with bursts of REM, coupled with skeletal muscle atonia [3,8-10]. The time spent in REM sleep increases over the length of the night and makes up 20% to 25% of TST [6]. Whilst REM sleep contributes to a restful night sleep, this phase has a lower threshold for awakening than SWS.

The onset of oneiric phenomena has traditionally been associated with REM sleep; however neurophysiological research indicates that dreaming occurs in both REM and NREM phases [11,12]. The occurrence of oneiric activity between the phases have some qualitative variances with dream activity during NREM sleep being associated with memory processing and recall of experiences, whilst in REM sleep it is characterised by visuohallucinatory and unusual content [13]. The role of dreaming is thought to have importance for cognition, associated with mental processing and memory consolidation.

#### Sleep disturbance in ICU

The sleep physiology of ICU patients indicates that whilst their TST is often normal (7 to 9 h) [2,14-17], sleep is highly fragmented with patients experiencing 6.2 awakenings per hour [2]. Sleep quality is further compromised as the majority of sleep is spent in stages N1 and N2 which is perceived to be ‘light sleep’ with limited restorative benefits. Subsequently, the characteristics of non-consolidated light sleep have resulted in ICU patients being sleep deprived. This has been attributed to a reduction in the duration of REM sleep and SWS, the supposition is that these phases support restorative processes and are important for recovery [16,18]. Due to a lack of quality sleep, patients experience daytime somnolence, with as much as 50% of patient TST occurring during daylight hours [15,16,19,20]. Sleep cycles which traverse both day and night perpetuate the phase shifting of circadian rhythms with sleep disturbance persisting months after discharge from ICU [21].

The impact of severity of illness on sleep architecture and sleep disturbance has been widely considered; however, there are limited studies explicitly investigating this factor. There exists some clinical indication that higher acuity scores are associated with greater sleep disturbance, specifically increased fragmentation and number of awakenings [22]. Gabor et al. [15] reported that healthy participants exposed to the ICU environment compared to ICU patients did not demonstrate the same level of sleep disturbance and reduction in SWS, suggesting that critical illness may be a contributing factor to the abnormalities seen in the sleep architecture of ICU patients. However, in this study, the healthy participants were only exposed to the noise within the environment and not to the full sensory experience of ICU such as frequent clinical interactions and invasive monitoring. Further, patients with higher acuity score also demonstrate considerable reduction in the EEG activity, which confounds the ability to accurately interpret polysomnographical data. Problematic to the investigation of severity of illness on sleep is the numerous contributing factors such as mechanical ventilation, pain, pharmacological agents and the greater need for clinical interventions, which confound the ability to determine causality.

#### Physiological effects of sleep disturbance

Physiologically, sleep deprivation has a myriad of negative cognitive, autonomic [23], metabolic [24,25] and hormonal [7,26,27] effects on the body that may contribute to prolonged ICU admission and in turn may contribute to an increase in patient morbidity. Findings derived from animal-based studies reveals that sleep deprivation is associated with increased mortality; the implications of such findings within the ICU setting have the capacity to be profound as sleep disturbance is commonly experience by this patient cohort [27,28].

#### Effects on the respiratory system

The chemosensitivity of the brain’s respiratory centre is reduced amongst sleep-deprived patients, with White et al. [29] reporting a decrease in intrinsic ventilatory response to hypoxic and hypercapnic states. Further, studies have reiterated varying adverse effects of sleep deprivation on respiratory muscle function including Phillip et al. [30] who demonstrated a significant reduction in FEV1 (mean decline of 6%) and FVC (mean decline of 5%) in sleep-deprived patients with underlying chronic obstructive pulmonary disease (COPD) compared to non-sleep-deprived COPD patients. Furthermore, maximal inspiratory pressure (MIP) was found to decrease from 81.5 ± 8.8 cmH_2_O in rested individuals to 75.9 ± 7.6 cmH_2_O in those who were sleep deprived [30]. Issues associated with inspiratory muscle endurance has also been reported by Cheng and Tang [31], who found that sleep deprivation amongst healthy individuals resulted in a significant decline in respiratory muscle strength. Findings such as these suggest that sleep-deprived states may contribute to patient hypoventilation, adversely effecting pulmonary reserves and overall readiness and ability to expedite weaning from mechanical ventilation.

#### Effects on the cardiovascular system

The manifestation of cardiovascular effects of sleep deprivation emanates from the stimulation of the sympathetic nervous system (SNS) and the release of the catecholamines adrenaline and noradrenaline. As a result, increases in blood pressure and heart rate are reported due to changes in baroreflex sensitivity [32-34]. Ogawa et al. [33] reported that increased baroreflex results in a 12 mmHg elevation in blood pressure as a result of a single night’s sleep deprivation amongst healthy individuals.

#### Effects on the immunological system

Neuro-immunological research asserts that there exists a bidirectional relationship between sleep deprivation and the immune system. Although the vast majority of studies conducted are animal based, the reported changes identified in the immunological system in response to sleep deprivation can have significant implications for critically ill patients.

The adaptive immune response and the functioning of the T cells have been reported to be vulnerable to sleep disturbance, as studies have identified that sleep plays an important role in the extravasation of the T cells [35,36]. The resulting increase in the propensity for sleep during illness may play an important role in promoting the function of the immune response and overall recovery. Previous animal studies have identified that interleukin (IL)-1 and tumour necrosis factor (TNF) contribute to an increase in non-REM sleep and sleep fragmentation [37]. This occurs as immunoreactive neurons of the cytokines which are located in regions of the brain that play a role in the sleep-wake cycle. Although the reported propensity for sleep during critical illness remains unknown, it is hypothesised that the role of IL-1 and its interaction with 5-hydrotryptamine (5-HT) may proffer an explanation. The onset of fever in response to invading pathogens potentiates the immune response and promotes survival. The resulting increase in pro-inflammatory cytokines such as IL-1 impacts on the sleep architecture through increasing the NREM phase with greater fragmentation and reducing REM sleep; these effects, however, allow for an enhanced inflammatory response [38-40]. The increased NREM sleep is thought to be an important aspect that supports the onset of fever; as during this phase the brain and body temperature is reduced, along with metabolic activity [37]. These features of the NREM stage allow for enhance heat dissipation with the corresponding increase in sleep fragmentation further facilitates heat loss [37].

Furthermore, the function of natural killer (NK) cells has been shown to be adversely effected in sleep-deprived states, although research findings have not always been consistent. Irwin et al. [41] found that the functionality of NK cells was reduced by 30% in sleep-deprived states in healthy subjects. This was further reiterated in their 1996 study which demonstrated that whilst NK cell activity initially declined, it did improve after sleep recovery. In contrast, Dinges et al. [42] reported that NK cell activity was increased in healthy adults who were sleep deprived; however, leukocyte function was suppressed. Although much of the research investigating the relationship between immune function and sleep deprivation has been conducted on healthy individuals, it is apparent that sleep provides an important role in the effective functioning of the immune system.

#### Effects on the metabolic system

During nocturnal sleep, downregulation occurs in the hypothalamus-pituitary-adrenal (HPA) axis and the SNS, which both have important roles in stress responses. Ensuing is a decrease in noradrenaline, adrenaline and cortisol levels which suppresses the immune systems anti-inflammatory cytokines [43]. During sleep, particularly SWS, growth hormone is released in combination with prolactin and leptin which provide pro-inflammatory signals which triggers the immune system activation [44]. The HPA axis plays an integral role in the body’s response to stress, primarily via the release of cortisol. Abnormal cortisol levels in ICU patients contribute to further disturbances with circadian rhythms, as cortisol release is normally increased prior to waking to stimulate the individual into a state ready to be active. An increase in cortisol levels and suppression of melatonin produces a propensity for patients to be in a wakeful state which may further exacerbate catabolic activity and increases oxygen consumption.

Sleep disturbance adversely effects carbohydrate metabolism resulting in insulin resistance and glucose clearance. Whereby, the increase in SNS activity and the ensuing stress response suppresses the release of insulin from pancreatic beta cells [45,46]. The impact of this on healthy individuals is an increased susceptibility to chronic health conditions such as coronary artery disease and diabetes. Within the ICU patient population, this can exacerbate underlying co-morbidities, putting them at risk for additional secondary complications. For example, instability in blood glucose levels has been associated with increased patient mortality [47]. The immunological effects of sleep disturbance also have a concomitant effect on metabolic functions with pro-inflammatory cytokines increasing in activity with moderate total sleep loss, which contributes to insulin resistance [44].

#### Effects on the neurocognitive system

The onset of delirium amongst ICU patient ranges between 60% to 80% for mechanically ventilated patients and 20% to 50% for non-mechanically ventilated patients [48,49]. Delirium has been identified as an independent predictor for adverse patient outcomes including increased hospital length of stay, a persistent decline in cognitive status and increased patient mortality [48-50].

A number of factors have been purported to contribute to the development of delirium, with sleep deprivation thought to be a contributing factor. However, the relationship is not clearly understood in regard to whether sleep deprivation leads to delirium or if the onset of delirium contributes to sleep deprivation. Nonetheless, sleep deprivation and delirium share a number of similar features namely a decline in cognitive function inclusive of psychomotor vigilance, memory and disturbances in language and perception which is characterised by hallucinations and delusions [51,52]. Although recommendations aimed at minimising the likely onset and complications associated with delirium include reducing sleep disturbance, one of the major challenges is associated with the pharmacological agents used to treat symptoms associated with delirium. Many of the pharmacological agents such as haloperidol and benzodiazepines used can potentially exacerbate sleep disturbance, due their inhibitory effect on SWS and REM sleep [52].

Commonly used GABA agonists such as propofol and benzodiazepines (Table 1) increase total sleep time and decrease sleep latency; however, these decrease N3 and REM sleep stages [53,54]. The pharmacokinetics of these medications suppresses cerebral function which produces changes in EEG activity such as a reduction in delta activity [55]. Similarly, commonly used opioids, morphine and fentanyl, increase total sleep time, they adversely impact N3 and inhibit REM sleep [56]. Although sedation and analgesia is commonly required to support therapies such as mechanical ventilation and promote patient comfort, understanding the cumulative effects of commonly used pharmacological agents and identifying newer emerging medications that may reduce the negative effects on sleep is an area for further research. Preliminary animal-based research regarding the effective of dexmedetomidine indicates that it increases N3 sleep, whilst ICU-based research suggests that incidences of delirium onset maybe reduced [57]. These preliminary findings suggest the dexmedetomidine may have an important role in the management of critically ill patients, which may reduce the incidences of delirium and the degree of sleep disturbance experience by patients.

**Table 1:** **The effect of pharmacological agents on sleep**

**Pharmacological agent**	**TST**	**SWS**	**REM**
Propofol	↑	↓	↓
Lorazepam	↑	↓	↓
Alprazolam	↑	↓	↓
Diazepam	↑	↓	↓
Haloperidol	↑	↑	↓
Morphine	↓	↓	↓
Fentanyl	↑	↓	↓
Midazolam	↑	↓	↓
Dexmedetomidine	**↑**	**↑**	**↓**

TST, total sleep time; SWS, slow wave sleep; REM, rapid eye movement; ↑, increase; ↓, decrease.

The impact that traumatic brain jury (TBI) has on sleep can be profound. The prevalence of TBI ranges between 30% and 84% and makes up a large percentage of critical care admissions [58,59]. In the acute phase of presentations, the impact that pharmacological agents combined with increased intra-cranial pressure can severely affect cerebral functioning and neurotransmitter activity resulting in decreased EEG activity. Sleep disturbance within this patient cohort may have significant implications for recovery outcomes, with previous research indicating that the presence of SWS and REM sleep, along with sleep efficiency correlated to improved cognitive outcomes [60]. The negative effects that TBI has on sleep can persist for months after ICU discharge and are predominantly associated with sleep initiation and maintenance and sleep architecture which can adversely affect cognition and behaviour [61,62].

#### Environmental factors impacting of ICU patients sleep

The importance of sleep in patient recovery can be deprioritized in ICU due to the need to meet increasingly complex care requirements. Sleep disturbance has emerged as an indicator for adverse clinical outcomes [52,63] and in turn should not be viewed as a passive physical state but rather acknowledged as an important bio-physiological process. The impact of the ICU environment on patient sleep has been frequently reported on and investigated as a primary etiological cause of sleep disturbance. Factors such as light, noise, clinical care-related interactions, combined with medications and acute illness have all been hypothesised as causal factors for sleep deprivation [1,2,7,15,16,19,64,65] (Table 2).

**Table 2:** **Factors impacting on sleep architecture**

**Factor**	**Example**
Environmental noise	Staff conversations and monitoring alarms
Prolonged exposure to low levels of artificial light	
Inflammatory mediators	
Pain	
Pharmacological agents	Sedative, opioids, benzodiazepines and inotropes
Mechanical ventilation	
Increased cortisol release	
Decreased endogenous melatonin levels	
Critical illness	
Clinical interactions	Vital sign monitoring, pathological investigations and medication administration

#### Effects of noise levels

Noise levels in ICU have been presumed to be the most disruptive environmental stressor that patients are subjected to and are a relentless feature due the cacophony of clinical activity and technological monitoring. Noise is defined in terms of mechanical energy and is commonly reported in the A weighted decibel (dB) scale as it most accurately attenuates the noise levels heard by the human ear [66]. The measure of decibels is logarithmic, and as a result, a 10-dB increase in noise constitutes a doubling of the noise level [67].

The World Health Organisation (WHO) recommends that noise levels within hospital wards should not exceed 30 dB(A) at night in order to reduce sleep disturbance [68]. The ability for ICUs to meet the WHO recommendations is improbable and is further reiterated within the literature whereby ICUs worldwide repeatedly cite noise levels in excess of 50 dB [2,64,65,69-71]. Moreover, noise levels in ICUs have steadily increased over the past decades, with a study conducted by Busch-Vishnich [72] quantifying this at a rate of 0.38 dB(A) per day and 0.42 dB(A) per night, per year. The evolving advances in medical care, technology and increasing patient acuity are contributing factors to the acoustical changes within the clinical environment. The noise generated from alarms associated with the intensive monitoring of patients frequently exceeds 70 dB(A), which is reflective of the noise generated by traffic [73]. Further, with the increasing ambient noise level, the impact of the Lombard effect can be compounded. This communication phenomenon results in individuals increasing their speech amplitude in order to enhance the intelligibility of their speech within noisy environments and contributes to a rapid escalation in environmental noise. This creates an environment that is detrimental to patients’ sleep as noise levels frequently undulate between low levels, with frequent short, sharp and unpredictable escalations in noise [74].

Both subjective and objective studies have reported noise as a primary source of sleep disturbance, with staff being the primary source of the generated noise [15,16,75]. Subjective studies investigating the impact of noise on sleep indicate that ICU patients identify the exposure to noise and inability to sleep as a major stressor recounted by patients. Further, objective studies indicate that the noise generated with within the environment is not conducive to the ability to sleep. The primary source of noise within the clinical environment has been attributed to staff behaviours, which Kahn et al. [76] reported accounted for greater than 50% of noise sources with an average noise level of 84 dB. Previous studies have suggested that the disruptive nature of staff behaviours evolve from the propensity of staff to deprioritized sleep, due to patients being intubated and sedated, and lacked knowledge regarding the psychophysiological effects of sleep deprivation [70,77]. As a result of subjective studies, behaviour modification strategies have been widely investigated as a means to curtail the impact of noise generated by staff on patient sleep and have been cited as being successful in reducing noise levels by 6% to 20% [78-80]. These behaviour modification activities have primarily involved an educational approach to enhance staff awareness of the impact their behaviours have in contributing to noise levels and promoting reductions in alarm volume settings to promote a diurnally appropriate environment. However, these studies have employed subjective assessments of the impact of behaviour modification, such as clinical staff perception of nocturnal noise, as opposed to using an objective environmental measure of noise. This has been problematic as studies that have employed environmental noise monitoring to determine the effect of behaviour modification on noise have been unable to achieve a measurable reduction in noise levels [81]. Furthermore, achieving sustainable change in the clinical environment has not been extensively investigated as behaviour modification studies have been undertaken over short durations, with follow-up assessments conducted after the immediate intervention period.

Studies employing objective measures of sleep have reported varying results. Aaron et al. [75] investigated the impact of noise on sleep via polysomnography (PSG) and reported that noise sources exceeding 50 dB(A) resulted in electroencephalographic changes indicative of arousal in 25% of cases. However, this finding has not been consistently reported in proceeding studies; further, there is a discrepancy between self-reported sleep quality and the biophysiological data obtained regarding sleep. Studies employing time synchronised monitoring of sleep via polysomnography and environmental noise monitoring, such as Freedman et al. [16] identified that only 11.5% of arousals and 17% of awakenings could be attributed to noise. Likewise, in a similar study, Gabor et al. [15] identified that 20% of awakenings were the direct result of environmental noise. Neither study was able to account for the cause of the remaining 75% to 80% of arousals but postulated that clinical interactions may account for much of these. However, this was not supported by Gabor et al.’s [15] findings, whereby only 7% of awakenings could be associated with clinical interactions. Subsequently, the degree to which noise acts as the pathogenesis of sleep disturbance in ICU remains unclear, although it is commonly reported as an issue by survivors of critical illness.

#### Effects of clinical interactions

The need for clinical interventions increases exponentially in ICU as many assessments are scheduled on an hourly basis such as vital sign monitoring, fluid balance assessments and pharmacological administration. These interventions in combination with basic care needs establish an environment in which sleep is easily deprioritized and disrupted. Furthermore, the design of ICU can compound issues, as the clinical area is frequently open planned with patients segregated from each other by curtains, which achieves little in quashing the impact of the clinical environment on patients’ senses.

Early observational studies conducted by Walker [82] and Woods [83] reported that patients were disturbed between 1 and 14 times per hour, and given that a single sleep cycle requires 90 to 110 min to complete, patients cared for in the ICU have little ability to achieve quality sleep. In more contemporary studies such as Lee et al.’s [84], 50% of subjects reported being woken 2 to 5 times throughout the night, which contributed to difficulties falling back to sleep once woken. This finding has been corroborated by previous studies conducted by Redeker and Hedges [85] and Tranmer et al. [1]. In contrast, studies conducted by Tamburri et al. [86] and Celik et al. [87] report much higher patient to staff interactions with both studies reporting between 40 and 60 interactions per night. Comparatively, the frequency of disruptions for mechanically ventilated patients was found to be substantially higher, with Le et al. [88] reporting that patients experienced 20 to 60 disruptions per hour.

The intensive monitoring that patients are subjected to is detrimental to the intrinsic sleep cycle, suggesting that under these conditions ICU patients will experience sleep deprivation. Further, in an observational study by Le et al. [88], nursing staff reported that 13.9% of nocturnal interactions could be safely omitted and, in turn, reduce the disturbance to patients. This suggests that a number of nursing-based activities are performed as a routine rather than based on clinical evidence and necessity. This highlights the need for clinical staff to critically evaluate the necessity for some of the care provided and to consider making adjustments to workflow in order to promote nocturnal sleep.

#### Effects of light

Regulation of the circadian rhythm is influenced by so-called zeitgebers (environmental cues) to remain entrained to the typical day/night routine. Prominent zeitgebers include meal times and exposure to daylight, both of which can be suppressed amongst critically ill patients. Nutrition is frequently provided enterally on a 24-h continuum, whilst ICU design and critical illness does not permit patient exposure to adequate natural light. Subsequently, the outcome of inappropriately timed zeitgebers in combination with prolonged exposure to low levels of artificial lighting can impact on the circadian pacemaker leading to circadian disturbance.

The protracted exposure to artificial lighting obfuscates the normal circadian rhythms, with light levels within ICUs being highly variable, ranging between a mean of 5 and 1,400 Lux. Exposure to low levels of artificial light less than 500 Lux and for as little as 20 min has been identified as sufficient to suppress nocturnal melatonin secretion [89-92]. Melatonin is an important regulator hormone of the circadian rhythm. Melatonin levels increase during the early hours of the night to facilitate the onset of sleep and decrease during the morning when cortisol levels increase. Whilst there is a focus on the potential role of melatonin in promoting sleep in this patient population, subjective studies have reported that ICU survivors perceived light exposure to be minimally disruptive to their ability to sleep [93]. Hu et al. [94] investigated the effect of non-invasive, non-pharmacological interventions of ear plugs and eye masks on ICU patients (*n* = 14) who underwent PSG sleep monitoring. The study found that these interventions resulted in an increase in recorded REM sleep, a reduction in REM latency with less arousals (*p* < 0.05) and an elevation in melatonin levels (*p* =0.002). Suggesting that although patients may not perceive exposure to light a major cause of sleep disturbance, it appears to have a detrimental effect of sleep wake patterns, and simple interventions may be able to minimise its effects.

A considerable body of research focuses on how light exposure affects endogenous melatonin secretion and the potential efficacy of supplemental melatonin as a means to entrain the circadian rhythm and overcome the lack of light variation to maintain the external zeitgeber. Munigler [95] reported that melatonin secretion was suppressed in patient with sepsis compared to non-septic ICU patients who demonstrated normalised circadian patterns similar to healthy individuals. In a double blind placebo controlled study (*n* = 8), patients receiving supplemental melatonin demonstrated an improvement in sleep duration and sleep quality [96]. In contrast, a study conducted by Egi et al. [97] involving 32 tracheostomised ICU patients was inconclusive regarding the benefits of melatonin in promoting sleep, finding that although melatonin levels increased in the treatment group it did not result in improved observable nocturnal sleep (240 min versus 243.4 min in the placebo group). A similar study undertaken by Bourne et al. [98] also studied the effect of supplemental nocturnal (21:00 h) melatonin administration of 10 mg in 27 ICU patients who had undergone trachesotomy insertion to promote ventilator weaning. The reported outcome of this study was that sleep in the treatment group increased by 1 h (sleep efficiency index difference = 0.12, 95% CI −0.02 to 0.27, *p* = 0.04) compared to the placebo group, along with an objective improvement in sleep quality as evaluated by bispectral index (BIS).

Melatonin has emerged as a possible treatment to maintain the circadian rhythm with oral administration having good bioavailability, with minimal negative effects on respiration despite its hypnotic effects [99]. To date, the studies conducted have involved small participant numbers and variances in sleep assessment. Future studies involving larger participant numbers and standardised sleep monitoring via PSG are required to ascertain the potential of melatonin as a standard therapy, along with the effect of concurrent interventions such as eye masks and ear plugs to negate some of the sensory experience of the ICU environment.

#### Sleep monitoring techniques

Sleep-related research conducted amongst ICU patients clearly identifies significant sleep disturbances, which can have deleterious implications on their psychophysiological condition which protract recovery. Despite these findings, there remains no method that is feasible for widespread implementation that accurately monitors patients’ sleep within the ICU environment. Although PSG provides considerable benefits over other strategies in being able to provide details of sleep physiology, it is not a strategy that is feasible to be implemented widely as it is expensive, labour intensive and technically difficult. In comparison, simple cost-effective methods involving clinical observations are questionable regarding their accuracy as studies have indicated that these result in an overestimation of sleep time and quality [17,65,100]. This was demonstrated by Nicolas and colleagues’ [8] quantitative single blinded descriptive study, investigating nursing-based assessment and patient perception of sleep which found that these correlated only 50% of the time, with nursing staff overestimating patients’ quality of sleep. Adjunct methods such as actigraphy (ACTG) have been considered, although studies involving the ICU are few and participant numbers are small. There are, however, some favourable attributes associated with ACTG use in comparison to PSG in that it is cost effective, is easy to interpret and is able to collect data over an extended period. The limitations of ACTG need to be acknowledged in that it will provide no information regarding the sleep architecture of patients and may be suitable only for a limited cohort of ICU patients. However, it may be potentially useful in monitoring circadian disturbance and sleep fragmentation, both of which are major issues associated with ICU patients. Potentially, further advancements in monitoring techniques and the development of advanced monitoring algorithms may provide clinicians with a viable method.

As a result there is a need to develop accurate and effective clinical monitoring methods of sleep, to facilitate the early recognition of sleep disturbance. Interventions need to be implemented that aim to provide an environment that supports sleep and recognises its role in patient recovery (Table 3). Additional research needs to be undertaken in order to understand the physiological impact of critical illness on patient sleep, the relationship between delirium and sleep disturbance, along with how mechanical ventilation can be utilised to facilitate nocturnal rest, and identification of pharmacological agents and regimes that do not interfere with the intrinsic sleep cycle. Further, identifying strategies that can be readily implemented that endeavour to minimise the impact of complications associated with sleep deprivation. Howbeit, research investigating sleep promotion strategies have lacked clinical efficacy [17,92,100], as PSG is not viable for widespread implementation, behavioural assessments such as ACTG have not been able to accurately assess sleep in critically ill patients, and subjective assessments such as nursing-based observation frequently overestimate sleep. Adjunct interventions such as behaviour modification of staff through education have only been subjectively evaluated and their impact on the long term is not evaluated. Emerging benefit of eye masks and ear plugs may have some benefit in quashing the effect of the ICU environment on patients but do improve the sleep architecture of patients. Similarly, melatonin administration reports some promising benefits; however, larger studies are required. A primary issue confronting clinicians regarding sleep in ICU is the development of a method that allows for sleep monitoring to be implemented as a standard of clinical care, which provides accurate information regarding the sleep of this patient cohort.

**Table 3:** **Interventions to reduce sleep disturbance**

**Intervention**	**Example**
Establish a nocturnal environment	By reducing volumes of alarms and phones.
Enhance staff awareness of noise produced by conversations	Soundear
Employ the use of night settings in monitoring equipment with backlighting to reduce exposure to light.	
Judicious use of pharmacological agents	
Monitor, assess and treat pain	
Devise individualised sleep hygiene routines	
Employ relaxation techniques in preparation for sleep	Music therapy and massage
Decrease environmental stimulus	Via the use of eye masks and ear plugs
Reduce nocturnal clinical interactions	Cluster clinical care and scheduling of procedures
Ventilator support to promote rest overnight	
Reduce daytime napping in order to reduce circadian dys-synchrony	
Promote daytime activity	Such as sitting out of bed and mobilisation

## Conclusions

Despite decades of research identifying the impact of the clinical environment on ICU patients’ sleep, little has been accomplished in overcoming the factors that are purported to contribute to sleep disturbance. There exists a multitude of factors that contribute to sleep disturbance that highlights the complexities faced when attempting to manage sleep disturbance and consequences of sleep deprivation on patient recovery. This is also inclusive of the intricate impact that severity of illness has on sleep which is yet to be fully elucidated. Clinicians involved in the provision of care to patients need to be attuned and aware of the consequences of sleep disturbance and how to make modifications to their environment and care planning in order to promote sleep and aid recovery. There exists a need to undertake further clinical research to identify effective strategies to curtail the impact of the clinical environment on patients’ ability to sleep and to enhance understanding between the impact of sleep deprivation and patient outcomes. Further, future research should be directed at identifying an accurate and feasible sleep monitoring method to facilitate the ability to implement strategies that endeavour to promote sleep and recovery, whilst decreasing the associated complications linked to sleep deprivation.

## Abbreviations

ACTG: actigraphy
BIS: bispectral index
COPD: chronic obstructive pulmonary disease
dB: decibels
HPA: hypothalamus-pituitary-adrenal axis
ICU: intensive care unit
IL: interleukins
Lux: unit of illuminance and luminous
MIP: maximal inspiratory pressure
N1: non-rapid eye movement stage 1 sleep
N2: non-rapid eye movement stage 2 sleep
N3: non-rapid eye movement stage 3 sleep
NK: natural killer cells
NREM: non-rapid eye movement
PSG: polysomnography
REM: rapid eye movement
SWS: slow wave sleep
TBI: traumatic brain injury
TNF: tumour necrosis factor
TST: total sleep time
